# Identification of high-performing antibodies for Moesin for use in Western Blot, immunoprecipitation, and immunofluorescence

**DOI:** 10.12688/f1000research.130126.2

**Published:** 2023-08-08

**Authors:** Walaa Alshafie, Riham Ayoubi, Maryam Fotouhi, Kathleen Southern, Carl Laflamme, NeuroSGC/YCharOS collaborative group

**Affiliations:** 1 Department of Neurology and Neurosurgery, Structural Genomics Consortium, The Montreal Neurological Institute, McGill University, Montreal, Quebec, H3A 2B4, Canada

**Keywords:** Uniprot ID P26038, MSN, Moesin, antibody characterization, antibody validation, Western Blot, immunoprecipitation, immunofluorescence

## Abstract

Moesin is a cytoskeletal adaptor protein, involved in the modification of the actin cytoskeleton, with relevance to Alzheimer’s Disease. Well characterized anti-Moesin antibodies would benefit the scientific community. In this study, we characterized ten commercial antibodies for Moesin in Western Blot, immunoprecipitation, and immunofluorescence using a standardized experimental protocol based on comparing read-outs in knockout cell lines and isogenic parental controls. We identified many high-performing antibodies and encourage readers to use this report as a guide to select the most appropriate antibody for their specific needs.

## Introduction

Moesin is a cytoskeletal adaptor protein that belongs to the Ezrin-Radixin-Moesin (ERM) family of proteins which connects the actin cytoskeleton to the plasma membrane, regulating the structure and function of specific domains of the cell cortex. ^1^^,^^2^ Moesin plays a pertinent role in immunity, acting on T and B-cell homeostasis and self-tolerance. ^3^^,^^4^ As such, specific mutations in this ERM protein have implications in immunodeficiency. ^5^^,^^6^ Studies have demonstrated that Moesin overexpression is associated with various cancer-related processes and can act as a prognostic marker. ^7^^–^^11^

Proteomic and protein co-expression network analysis of Alzheimer's Disease (AD) brain has revealed a module that is enriched in inflammation-related proteins. ^12^ Moesin, along with CD44 antigen, have emerged as key drivers in this inflammation module. Disrupting the Moesin-CD44 pathway is a current focus in AD research. ^13^ Mechanistic studies would be greatly facilitated with the availability of high-quality antibodies.

Here, we compared the performance of a range of commercially available antibodies for Moesin and identified high-performing antibodies for Western Blot, immunoprecipitation and immunofluorescence, enabling biochemical and cellular assessment of Moesin properties and function.

## Results and discussion

Our standard protocol involves comparing readouts from wild-type and knockout cells. ^14^^,^^15^ The first step is to identify a cell line(s) that expresses sufficient levels of a given protein to generate a measurable signal. To this end, we examined the DepMap transcriptomics database to identify all cell lines that express the target at levels greater than 2.5 log _2_ (transcripts per million “TPM” +1), which we have found to be a suitable cut-off (Cancer Dependency Map Portal, RRID:SCR_017655). Commercially available HeLa cells expressed the Moesin transcript at RNA levels above the average range of cancer cells analyzed. Parental and *MSN* knockout HeLa cells were obtained from Abcam ( Table 1).

**Table 1.** Summary of the cell lines used.

Institution	Catalog number	RRID (Cellosaurus)	Cell line	genotype
Abcam	ab255448	CVCL_0030	HeLa	WT
Abcam	ab265020	CVCL_B9VN	HeLa	*MSN* KO

**Figure 1.**
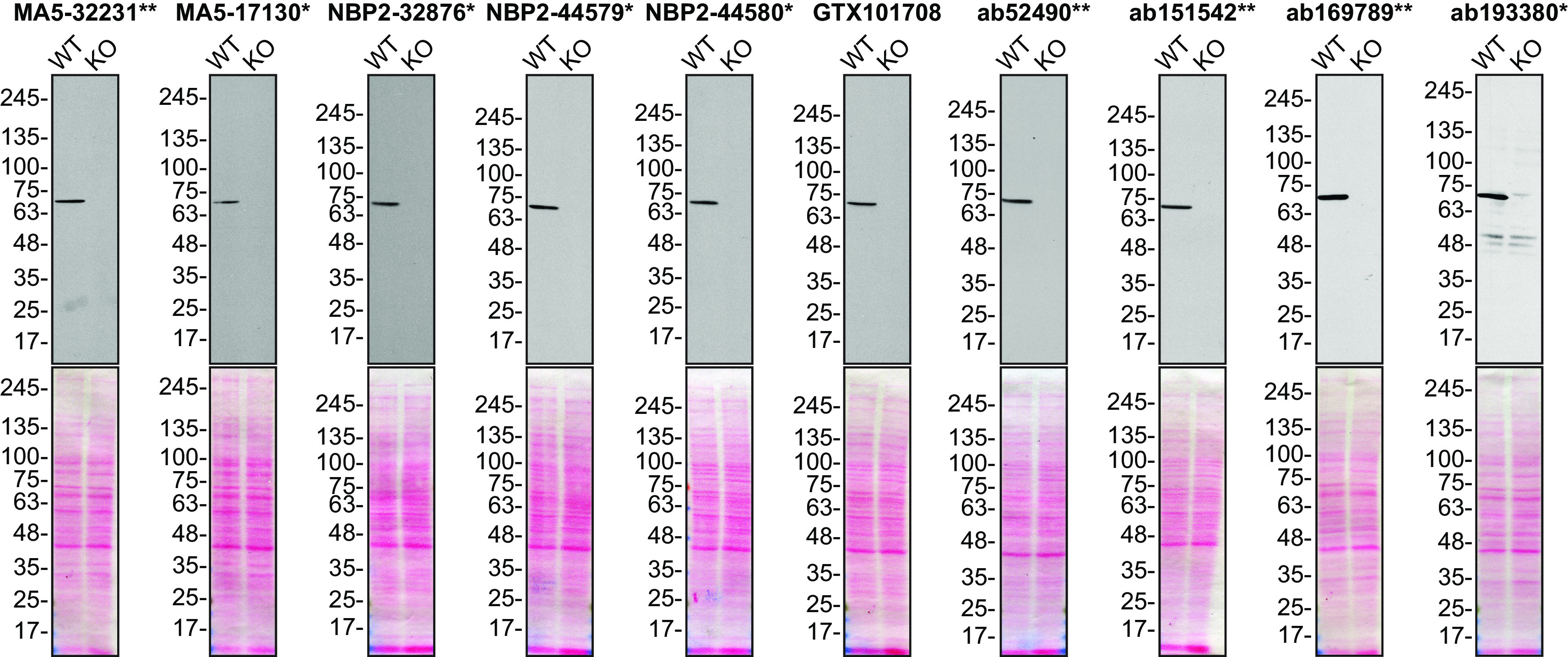
Moesin antibody screening by Western Blot. Lysates of HeLa (WT and *MSN* KO) were prepared, and 25 μg of protein were processed for Western Blot with the indicated Moesin antibodies. The Ponceau stained transfers of each blot are presented to show equal loading of WT and KO lysates and protein transfer efficiency from the acrylamide gels to the nitrocellulose membrane. Antibody dilutions were chosen based on the recommendations provided by suppliers with exceptions for antibodies MA5-32231**, MA5-17130* and GTX101708, which were titrated as the signal received was too strong following the supplier’s recommendations. The antibody dilutions were as follows: MA5-32231** at 1/5000, MA5-17130* at 1/5000, NBP2-32876* at 1/5000, NBP2-44579* at 1/5000, NBP2-44580* at 1/5000, GTX101708 at 1/5000, ab52490** at 1/1000, ab151542** at 1/1000, ab169789** at 1/10000 and ab193380** at 1/400. Predicted band size: 68 kDa. *Monoclonal antibody, **Recombinant antibody.

For Western Blot, we resolved proteins from wild-type and *MSN* KO cell extracts and probed them side-by-side with all antibodies in parallel ( Figure 1).

**Figure 2.**
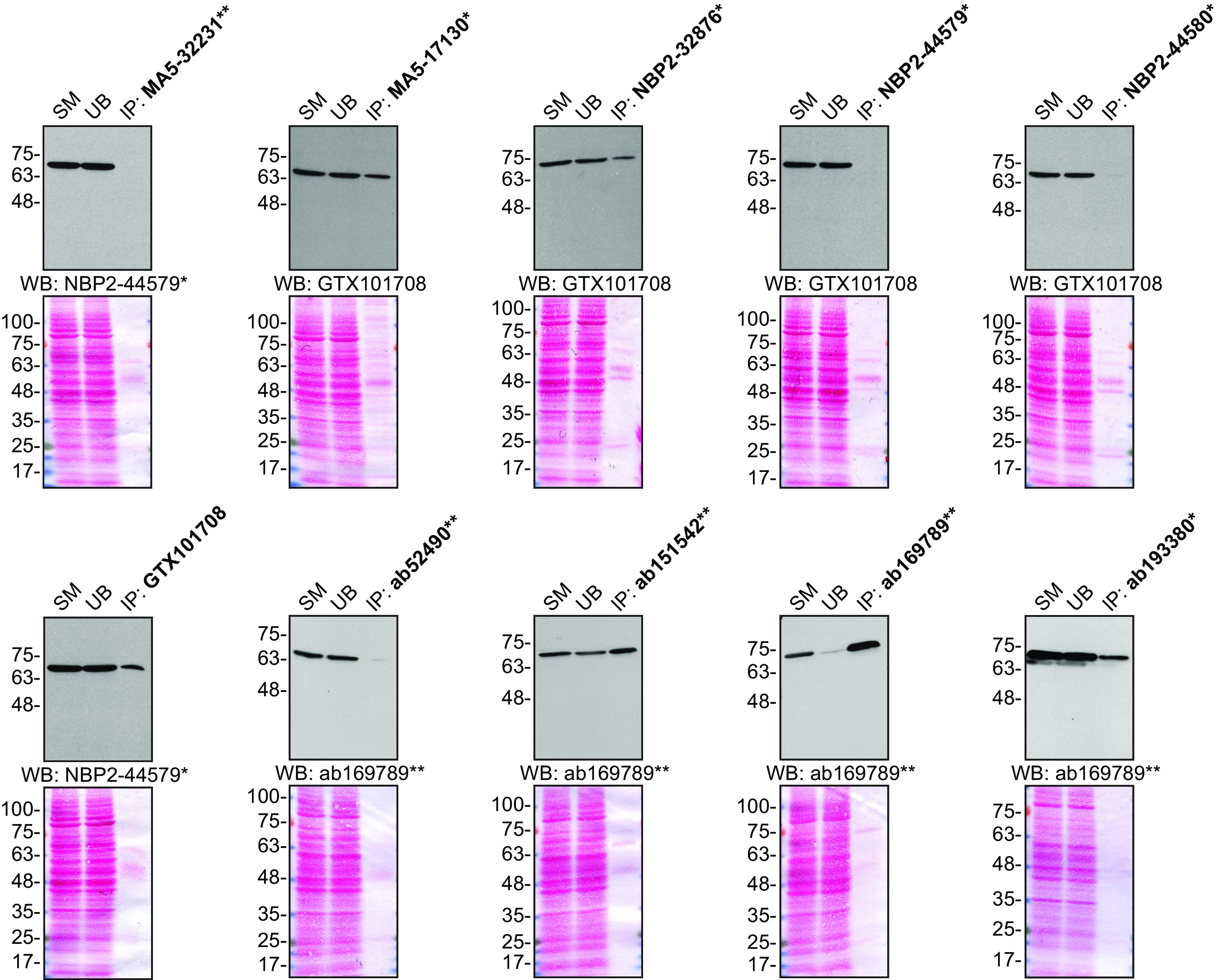
Moesin antibody screening by immunoprecipitation. HeLa lysates were prepared, and IP was performed using 1.0 μg of the indicated Moesin antibodies pre-coupled to either protein G or protein A Sepharose beads. Samples were washed and processed for Western Blot with the indicated Moesin antibody. For immunoblot, NBP2-44579*, GTX101708 and ab169789** were used at 1/20000, 1/20000 and 1/10000, respectively. The Ponceau stained transfers of each blot are shown for similar reasons as in Figure 1. SM = 10% starting material; UB = 10% unbound fraction; IP = immunoprecipitate. *Monoclonal antibody, **Recombinant antibody.

For immunoprecipitation, we used the antibodies to immunopurify Moesin from HeLa cell extracts. The performance of each antibody was evaluated by detecting the Moesin protein in extracts, in the immunodepleted extracts and in the immunoprecipitates ( Figure 2).

**Figure 3.**
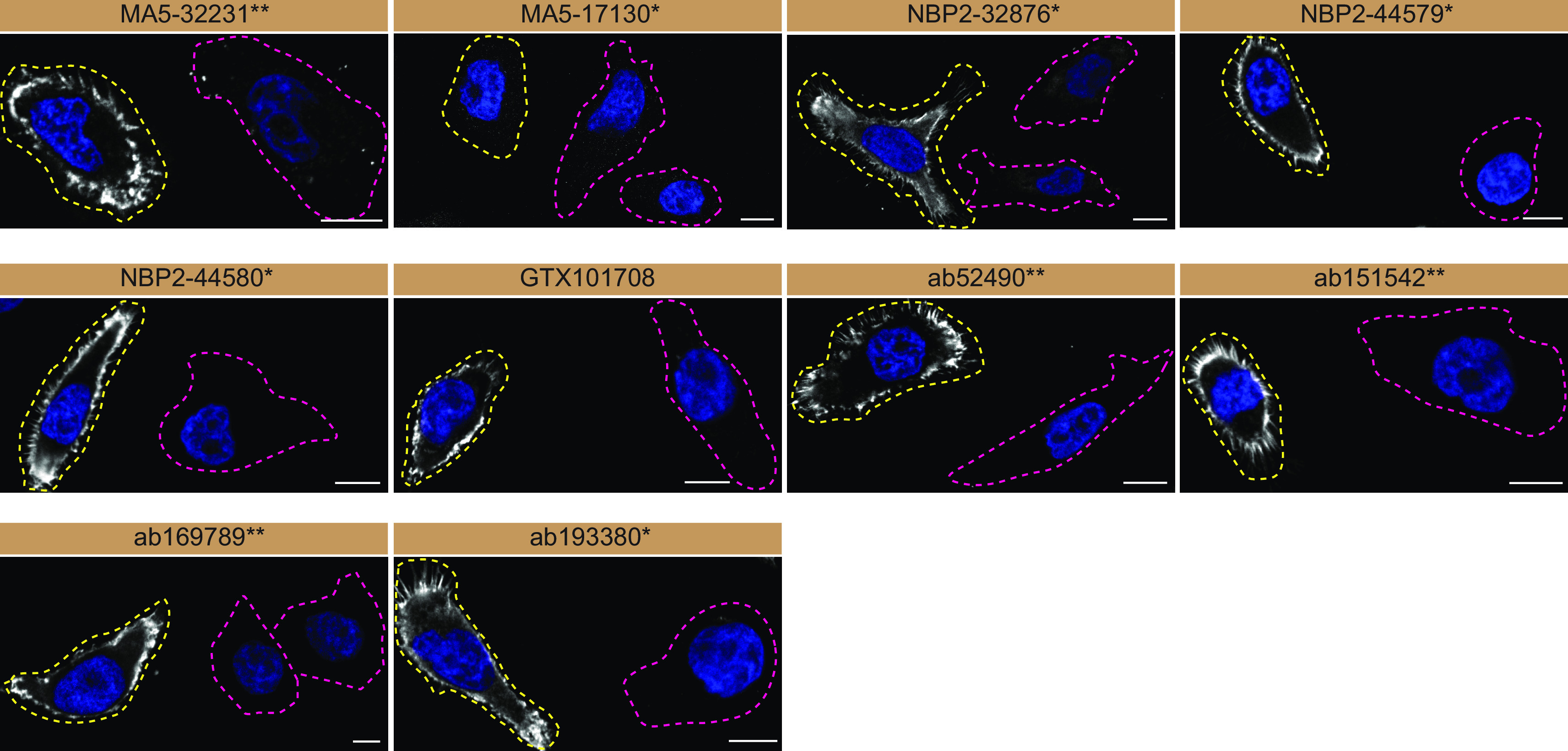
Moesin antibody screening by immunofluorescence. HeLa WT and *MSN* KO cells were labelled with a green or a far-red fluorescent dye, respectively. WT and KO cells were mixed and plated to a 1:1 ratio on coverslips. Cells were stained with the indicated Moesin antibodies and with the corresponding Alexa-fluor 555 coupled secondary antibody including DAPI. Acquisition of the blue (nucleus-DAPI), green (WT), red (antibody staining) and far-red (KO) channels was performed. Representative images of the merged blue and red (grayscale) channels are shown. WT and KO cells are outlined with yellow and magenta dashed line, respectively. Antibody dilutions were chosen based on supplier recommendations, except for MA5-32231** which was titrated to 1/1000 as the signal was too strong. When concentrations were not provided by the supplier, antibodies were tested at 1/1000, which was the case for MA5-17130*. Antibody dilutions used; MA5-32231** at 1/1000; MA5-17130* at 1/1000; NBP2-32876* at 1/200; NBP2-44579* at 1/200; NBP2-44580* at 1/200; GTX101708 at 1/200; ab52490** at 1/200, ab151542** at 1/200, ab169789** at 1/100, ab193380** at 1/200. Bars = 10 μm. *Monoclonal antibody, **Recombinant antibody.

For immunofluorescence, as described previously, antibodies were screened using a mosaic strategy. ^16^ In brief, we plated WT and KO cells together in the same well and imaged both cell types in the same field of view to reduce imaging and analysis bias ( Figure 3).

In conclusion, we have screened Moesin commercial antibodies by Western Blot, immunoprecipitation and immunofluorescence, and identified several high-quality antibodies under our standardized experimental conditions.

## Methods

### Antibodies

All Moesin antibodies are listed in Table 2. Peroxidase-conjugated goat anti-rabbit and anti-mouse antibodies are from Thermo Fisher Scientific (cat. number 65-6120 and 62-6520). Alexa-555-conjugated goat anti-rabbit and anti-mouse secondary antibodies are from Thermo Fisher Scientific (cat. number A21429 and A21424).

**Table 2.** Summary of Moesin antibodies tested.

Company	Catalog number	Lot number	RRID (Antibody Registry)	Clonality	Clone ID	Host	Concentration (μg/μl)	Vendors recommended applications
Thermo Fisher Scientific	MA5-32231**	VJ3101165	AB_2809517	recombinant-mono	SC69-01	rabbit	1.00	Wb, IF
Thermo Fisher Scientific	MA5-17130*	VJ3101185	AB_2538601	monoclonal	2C12	mouse	1.00	Wb
Novus Biologicals (a Bio-Techne brand)	NBP2-32876*	4478-1XP160531	AB_2885048	monoclonal	SPM562	mouse	0.20	Wb, IF
Novus Biologicals (a Bio-Techne brand)	NBP2-44579*	44578-2P190315	AB_2885047	monoclonal	MSN/492	mouse	0.20	Wb, IF
Novus Biologicals (a Bio-Techne brand)	NBP2-44580*	4478-3P190605	AB_2885046	monoclonal	MSN/493	mouse	0.20	Wb, IF
GeneTex	GTX101708	40198	AB_10618789	polyclonal	-	rabbit	0.21	Wb, IP, IF
Abcam	ab52490**	GR3207377-11	AB_881245	recombinant-mono	EP1863Y	rabbit	0.20	Wb, IP, IF
Abcam	ab151542**	GR112662-8	AB_2893185	recombinant-mono	EPR2428(2)	rabbit	0.09	Wb, IF
Abcam	ab169789**	GR121830-3	AB_2885098	recombinant-mono	EPR2429(2)	rabbit	0.07	Wb, IP, IF
Abcam	ab193380*	GR3373113-1	AB_2885109	monoclonal	MSN/491	mouse	0.20	Wb, IP

Wb = Western blot; IF = immunofluorescence; IP = immunoprecipitation.

### Cell culture

HeLa WT and *MSN* KO cells used are listed in Table 1. Cells were cultured in DMEM high-glucose (GE Healthcare cat. number SH30081.01) containing 10% fetal bovine serum (GE Healthcare cat. Number SH30072.03), 2 mM L-glutamate (Wisent cat. number 609065, 100 IU penicillin and 100 μg/ml streptomycin (Wisent cat. number 450201).

### Antibody screening by Western Blot

Western Blots were performed as described in our standard operating procedure. ^17^ HeLa WT and *MSN* KO cells were collected in RIPA buffer (50 mM Tris pH 8.0, 150 mM NaCl, 1.0 mM EDTA, 1% Triton X-100, 0.5% sodium deoxycholate, 0.1% SDS) supplemented with protease inhibitor. Lysates were sonicated briefly and incubated 30 min on ice. Lysates were spun at ~110,000 × g for 15 min at 4°C and equal protein aliquots of the supernatants were analyzed by SDS-PAGE and Western Blot.

Western Blots were performed with large 4–15% gradient polyacrylamide gels and transferred on nitrocellulose membranes. Proteins on the blots were visualized with Ponceau staining which is scanned to show together with individual Western Blot. Blots were blocked with 5% milk for 1 hr, and antibodies were incubated O/N at 4°C with 5% bovine serum albumin in TBS with 0.1% Tween 20 (TBST). Following three washes with TBST, the peroxidase conjugated secondary antibody was incubated at a dilution of ~0.2 μg/ml in TBST with 5% milk for 1 hr at room temperature followed by three washes with TBST. Membranes are incubated with ECL from Pierce (cat. number 32106) prior to detection with HyBlot CL autoradiography films from Denville (cat. number 1159T41).

### Antibody screening by immunoprecipitation

Immunoprecipitation was performed as described in our standard operating procedure. ^18^ Antibody-bead conjugates were prepared by adding 1.0 μg of antibody to 500 μl of PBS with 0.01% triton X-100 in a microcentrifuge tube, together with 30 μl of protein A - (for rabbit antibodies) or protein G - (for mouse antibodies) Sepharose beads. Tubes were rocked overnight at 4°C followed by two washes to remove unbound antibodies.

HeLa cells were collected in HEPES buffer (20 mM HEPES, 100 mM sodium chloride, 1 mM EDTA, 1% Triton X-100, pH 7.4) supplemented with protease inhibitor. Lysates are rocked 30 min at 4°C and spun at 110,000 × g for 15 min at 4°C. One ml aliquots at 1 mg/ml of lysate were incubated with an antibody-bead conjugate for ~2 hrs at 4°C. Following centrifugation, the unbound fractions were collected, and beads were subsequently washed three times with 1.0 ml of HEPES lysis buffer and processed for SDS-PAGE and Western Blot on a 4-15% acrylamide gel.

### Antibody screening by immunofluorescence

Immunofluorescence was performed as described in our standard operating procedure. ^16^ HeLa cells (WT and MSN KO) were labelled with a green dye and with a deep red fluorescent dye from Abcam (cat. number ab176735 and ab176736), respectively. WT and KO cells were plated on glass coverslips as a mosaic and incubated for 24 hrs in a cell culture incubator. Cells were fixed in 4% PFA (in PBS) for 15 min at room temperature and then washed 3 times with PBS. Cells were permeabilized in PBS with 0.1% Triton X-100 for 10 min at room temperature and blocked with PBS with 5% BSA, 5% goat serum and 0.01% Triton X-100 for 30 min at room temperature. Coverslips were incubated face down on a 50 μl drop (on paraffin film in a moist chamber) with IF buffer (PBS, 5% BSA, 0,01% Triton X-100) containing the primary Moesin antibodies O/N at 4°C. Cells were washed 3 times for 10 min with IF buffer and incubated with corresponding Alexa Fluor 555-conjugated secondary antibodies, including DAPI, in IF buffer at a dilution of 1.0 μg/ml for 1 hr at room temperature. Cells were washed 3 times for 10 min with IF buffer and once with PBS. Coverslips were mounted on a microscopic slide using fluorescence mounting media (DAKO).

Imaging was performed using a Zeiss LSM 880 laser scanning confocal microscope equipped with a Plan-Apo 40× oil objective (NA = 1.40). Analysis was done using Image J. All cell images represent a single focal plane. Figures were prepared using Adobe Photoshop to adjust contrast, apply 1 pixel Gaussian blur and then assembled with Adobe Illustrator.

## Data Availability

Zenodo: Antibody Characterization Report for Moesin, DOI: https://doi.org/10.5281/zenodo.4724169. ^19^ Zenodo: Dataset for the Moesin antibody screening study, DOI: https://doi.org/10.5281/zenodo.7566164. ^20^ This project contains the following data:
- Head to head comparison of available commercial antibodies against Moesin by immunoblot (Western blot), immunoprecipitation and immunofluorescence. - This project contains the following underlying data included in a study aiming at characterizing antibodies for the Moesin protein. The study is available on Zenodo ( https://doi.org/10.5281/zenodo.4724169). ^19^ Data are available under the terms of the Creative Commons Attribution 4.0 International license (CC-BY 4.0).
