# Supplementary material for: Identification of high-performing antibodies for Moesin for use in Western Blot, immunoprecipitation, and immunofluorescence
Source: F1000Res. 2023 Dec 1;12:172. Originally published 2023 Feb 13. [Version 3] doi: 10.12688/f1000research.130126.3 (PMC10724652; doi:10.12688/f1000research.130126.3)
Supplement: Peer review history

# Reviewer report: Reviewer response for version 3

**DOI:** 10.5256/f1000research.159517.r227712

**Published:** 2023-12-15

**Authors:** Yosuke Senju, Emi Hibino

**Affiliations:** 1 Research Institute for Interdisciplinary Science (RIIS), Okayama University, Okayama, Japan; 2 Department of Basic Medicinal Sciences Graduate School of Pharmaceutical Sciences, Nagoya University, Aichi, Japan

The authors have addressed the concerns.

Are sufficient details of methods and materials provided to allow replication by others?

Yes

Is the rationale for creating the dataset(s) clearly described?

Yes

Are the datasets clearly presented in a useable and accessible format?

Partly

Are the protocols appropriate and is the work technically sound?

Yes

Reviewer Expertise:

biophysics, synthetic biology

We confirm that we have read this submission and believe that we have an appropriate level of expertise to confirm that it is of an acceptable scientific standard.

# Reviewer report: Reviewer response for version 2

**DOI:** 10.5256/f1000research.154210.r220232

**Published:** 2023-11-15

**Authors:** Yosuke Senju, Emi Hibino

**Affiliations:** 1 Department of Basic Medicinal Sciences Graduate School of Pharmaceutical Sciences, Nagoya University, Aichi, Japan; 2 Research Institute for Interdisciplinary Science (RIIS), Okayama University, Okayama, Japan

Moesin is vital for linking the actin cytoskeleton to the plasma membrane to stabilize the cell cortex, and is related to several diseases. Thus, it is essential to characterize moesin-specific antibodies. In this study, the authors characterized moesin-specific commercially available antibodies for western blotting, immunoprecipitation, and immunofluorescence using *MSN* knockout cell lines. This study will help readers to select the appropriate antibodies according to their applications.

 To make the article scientifically sounds, the following points should be addressed.

**Major comments:**

**Figure 1:** Regarding the ab52490 antibody, the author loaded 20 ug of protein for western blot in the company's webpage, while they described 25 ug in this manuscript.

**Figures 1 and 3:** The ab193380 antibody is described as a monoclonal antibody (*) in Table 2, while it is described as a recombinant antibody (**) in Figures 1 and 3. 

**Figures 1 and 3:** Regarding the NBP2-32876, NBP2-44579, NBP2-44580 antibodies (Bio-Techne), the authors used U2OS cells for the same western blot and immunofluorescence images in the company's webpage, while they described using HeLa cells in this manuscript. 

**Figure 2:** Is there any reason why the vendor recommends IP for ab52490, and does not recommend for MA5-17130 and NBP2-32876 in Table 2?

**Figure 3:** Is there any reason why the vendor does not recommend IF for ab193380 in Table 2?

**Minor comments:**

**Figure 3:** yellow and magenta dashed "line" should be yellow and magenta dashed "lines".

 Please carefully check citations, figure labels, format, language, and readability once more.

 I would like to thank the authors for their efforts to promote open science.

Are sufficient details of methods and materials provided to allow replication by others?

Yes

Is the rationale for creating the dataset(s) clearly described?

Yes

Are the datasets clearly presented in a useable and accessible format?

Partly

Are the protocols appropriate and is the work technically sound?

Yes

Reviewer Expertise:

biophysics

We confirm that we have read this submission and believe that we have an appropriate level of expertise to confirm that it is of an acceptable scientific standard, however we have significant reservations, as outlined above.

# Response

**Published:** 2023-11-28

**Authors:** Kathleen Southern

**Affiliations:** McGill University, Canada

Thank you Emi Hibino and Yosuke Senju for your peer-review report on this guide to selecting high-performing antibodies for Moesin in three common applications.

 The following changes have been made in response to your comments regarding Figure 1 and 3:

1. ab193380 is in fact a monoclonal antibody and thus the second star has been removed
2. “yellow and magenta dashed line” has been changed to “yellow and magenta dashed lines”

Thank you for being observant and catching these errors! A new version of this publication has been submitted to F1000 which include the changes above.

 Please see our responses below regarding your major comments:

***Figure 1:** Regarding the ab52490 antibody, the author loaded 20 ug of protein for western blot in the company's webpage, while they described 25 ug in this manuscript.*

 When performing the Western Blot, each lane of the gel is loaded with equal amounts of protein. That is why we couldn’t make an exception for this specific antibody as we have to have equal amounts of protein in order to correctly compare the antibodies performance side by side.

***Figures 1 and 3:** Regarding the NBP2-32876, NBP2-44579, NBP2-44580 antibodies (Bio-Techne), the authors used U2OS cells for the same western blot and immunofluorescence images in the company's webpage, while they described using HeLa cells in this manuscript.*

 As described in our results & discussion section, we select cell lines from based on public transcriptomic database, DepMap. The cell lines chosen are based on transcript level cut-off of the protein of interest. Based on the expression datasheet for Moesin, available on DepMap, HeLa cells expressed higher RNA levels of MSN compared to U2OS, which is why HeLa WT and MSN KO cells were obtained. 

***Figure 2:** Is there any reason why the vendor recommends IP for ab52490, and does not recommend for MA5-17130 and NBP2-32876 in Table 2?*

 The vendors recommend applications column is prior to validation by YCharOS. Once the validation data for Wb, IP and IF is collected, it is shared with the antibody manufacturer partners. Based on the results, it is then up the discretion of each companies whether or not they change the vendor recommended applications on their website. If you were to visit the vendor’s website for antibodies MA5-17130 and NBP2-32876, you’d see that they have included IP in their recommended applications based on the characterization data provided by YCharOS. Antibody ab52490, although was not successful in IP was not removed by the supplier.

***Figure 3:** Is there any reason why the vendor does not recommend IF for ab193380 in Table 2?*

 As mentioned previously, the list of vendor recommended applications in Table 2 is based on the vendors website, prior to the availability of validation data provided by YCharOS.  The vendors did not know prior to YCharOS testing that this antibody was successful in IF. Regardless, the vendors have decided to discontinue this specific antibody from their catalog.

 Thank you again for this thorough and detailed report. We hope we addressed all of your concerns and that the article meets your standards to be approved.

# Reviewer report: Reviewer response for version 1

**DOI:** 10.5256/f1000research.142858.r177143

**Published:** 2023-08-02

**Authors:** Sungsoo Na

**Affiliations:** 1 Indiana University Purdue University Indianapolis, Indianapolis, Indiana, USA

This manuscript compared the commercially available antibodies through various assays, including Western blot, immunoprecipation, and immunofluorescence. All the experimental methods and data are well presented. However, this manuscript lacks descriptions on the comparison, characterization, and identification of the antibodies in the results section although the authors claimed that this manuscript is a "guide to select the most appropriate antibody for their specific needs".

Are sufficient details of methods and materials provided to allow replication by others?

Partly

Is the rationale for creating the dataset(s) clearly described?

Yes

Are the datasets clearly presented in a useable and accessible format?

Partly

Are the protocols appropriate and is the work technically sound?

Yes

Reviewer Expertise:

live cell imaging, cell signaling analysis

I confirm that I have read this submission and believe that I have an appropriate level of expertise to confirm that it is of an acceptable scientific standard, however I have significant reservations, as outlined above.

# Response

**Published:** 2023-08-03

**Authors:** Kathleen Southern

**Affiliations:** McGill University, Canada

Thank you Sungsoo Na for reviewing this article.

 As a third-party entity, YCharOS refers to remain unbiased which is why the authors do not compare the performance of antibodies based on the displayed results. After presenting the YCharOS initiative on several occasions we have found that, for the most part, scientists interested in our reports have the expertise to interpret the results and decipher which antibodies are appropriate for their research needs. Furthermore, these antibodies are tested under one specific condition and scoring the antibodies would only be valid under this setup and cell line used. We believe that the results from Western Blot, immunoprecipitation and immunofluorescence are telling and will help viewers in finding high-quality antibodies for Moesin. For those that have trouble interpreting the results, we have a clear description on what a “successful” antibody looks like in each application on our FAQ page of the YCharOS gateway ( https://f1000research.com/gateways/ycharos/faqs ).

 We hope you understand our reasoning for our wish to remain unbiased. If there are any other changes you believe would be appropriate to enhance this manuscript that do not involve comparing or scoring the antibodies based on performance, please let us know and we would be happy to do so.

 Thank you again for your feedback.

# Reviewer report: Reviewer response for version 1

**DOI:** 10.5256/f1000research.142858.r177135

**Published:** 2023-08-02

**Authors:** Lyndsay Avery

**Affiliations:** 1 Biology, Saint Michael's College, Colchester, Vermont, UK

In this manuscript, the authors aim to validate 10 commercially available moesin antibodies for western blot, immunoprecipitation, and immunofluorescence. This work makes evident that there is great variability in both what each antibody is suited for and the conditions in which it can be used. In most cases, the vendor has not included some potential uses for the antibody as validated by the authors. But in some, the vendors recommendations don’t work well for the tested application.  

 Suggestions for improvement:

- In introducing the work, the authors point out that moesin is implicated in inflammation seen in Alzheimer’s Disease. However, they neglect to introduce its role in other important diseases such as immunodeficiency and cancer, among many others. A more thorough introduction with a more recent literature search would make this paper searchable for many scientists who are interested in these data.
- It's unclear how the antibodies were chosen for these assays. There are many commercially available moesin antibodies. It’s important to include more cited antibodies in the analysis such as those from Cell Signaling Technologies (CST) and Developmental Studies Hybridoma Bank (DSHB) as these are significantly cheaper than the antibodies currently assessed. Nonetheless, this analysis is informative for research scientists across many fields.
- It should be noted that the Biotechne antibodies are available through Novus Biologicals. While Novus is a Biotechne company, the antibodies are not searchable on their site.
- For immunoprecipitation data, a negative control for non-specific binding is important. The antibodies that positively IP in WT HeLa cells should be assessed in the MKO HeLa cells.
- Flow cytometry is a vendor recommended application for many of these antibodies. If possible, adding this to the analysis would strengthen the results.
- One concern is that one moesin antibody from Abcam (registry # AB_2885109) has since been discontinued by the manufacturer as they found that it was not specific to moesin. This antibody should be removed from analysis or addressed as nonspecific in the discussion.

Are sufficient details of methods and materials provided to allow replication by others?

Yes

Is the rationale for creating the dataset(s) clearly described?

Partly

Are the datasets clearly presented in a useable and accessible format?

Yes

Are the protocols appropriate and is the work technically sound?

Yes

Reviewer Expertise:

Immunology, T cell biology, migration, cytoskeleton, hematopoiesis

I confirm that I have read this submission and believe that I have an appropriate level of expertise to confirm that it is of an acceptable scientific standard, however I have significant reservations, as outlined above.

# Response

**Published:** 2023-08-03

**Authors:** Kathleen Southern

**Affiliations:** McGill University, Canada

Thank you, Lyndsay Avery, for your response, your feedback is much appreciated.

 We will be submitting a new version of the manuscript with your following suggestions included. In the introduction, we have included more recent information on Moesin’s implications in disease. As for Table 2, we have made it clear that the Bio-Techne antibodies are available from Novus Biologicals, which is a Bio-Techne brand.

 As for your point regarding how the antibodies were chosen for the assays, the antibodies to be tested are donated by YCharOS partners ( https://ycharos.com/partners/), who cover nearly 28% of all antibodies listed on the Antibody registry ( antibodyregistry.org). When the industry partners are informed of upcoming targets, they donate the antibodies they have the capacity to supply at the time the study commences. At the time this study for Moesin was performed, we did not yet establish partnerships with CST nor DHSB, which is why their antibodies were not tested. It would be a great to characterize these antibodies as a follow-up study.

 To respond to your comment regarding a negative control for immunoprecipitation, we too agree that negative controls to demonstrate any non-specific binding are important when validating antibodies by immunoprecipitation. That being said, under our protocol, all antibodies were tested under the same conditions within the same experiment, any antibody that did not show an enrichment in the immunoprecipitate acts as the negative control.

 As majority of researchers seem to be more interested in protein localization rather than quantifying the presence of a protein in a cell, we have prioritized testing the antibodies under immunofluorescence and have included it in our standard operating procedure. Nonetheless, flow cytometry is a very useful application that will be considered as a follow-up study in the future.

 To respond to your last point, Abcam made the decision to remove antibody AB_2885109 based on the result of this study and was discontinued thereafter as to provide their customers with highest quality standards of products. For more information as to why the antibody was discontinued, you can visit their website ( https://www.abcam.com/products/primary-antibodies/moesin-antibody-msn491-ab193380.html). We believe it’s important to demonstrate that our industry partners trust our validation procedure and use it as an opportunity to re-evaluate the quality of their products, which is why we kept antibody AB_2885109 in the results.

 We hope you understand our reasoning for not making all of your suggested changes and are happy to receive further feedback!
